# Adverse Birth Outcomes Associated with Heat Stress and Wildfire Smoke Exposure During Preconception and Pregnancy

**DOI:** 10.1021/acs.est.4c10194

**Published:** 2025-06-18

**Authors:** Roxana Khalili, Yisi Liu, Yan Xu, Karl O’Sharkey, Nathan Pavlovic, Crystal McClure, Fred Lurmann, Tingyu Yang, Xinci Chen, Mario Vigil, Brendan Grubbs, Layla Al Marayati, Deborah Lerner, Nathana Lurvey, Carmen J. Marsit, Jill Johnston, Theresa M. Bastain, Carrie V. Breton, Shohreh F. Farzan, Rima Habre

**Affiliations:** † Department of Population and Public Health Sciences, Keck School of Medicine, 5116 University of Southern California, Los Angeles, California 90032, United States; ‡ 375568 Sonoma Technology, Inc., Petaluma, California 94954, United States; § Department of Obstetrics and Gynecology, Keck School of Medicine, University of Southern California, Los Angeles, California 90033, United States; ∥ 431513 Eisner Health, Los Angeles, California 90015, United States; ⊥ Gangarosa Department of Environmental Health, Rollins School of Public Health, 1371 Emory University, Atlanta, Georgia 30322, United States; # Spatial Sciences Institute, Los Angeles, California 90089, United States

**Keywords:** wildfire smoke, heat stress, pregnancy, preconception, birth outcomes, growth, climate vulnerability

## Abstract

We investigated associations between preconception and prenatal heat stress and wildfire (WF) smoke exposures on adverse birth outcomes and whether neighborhood climate vulnerability is an effect modifier in the Maternal And Developmental Risks from Environmental and Social stressors cohort (*N* = 713). Generalized linear models were fit to test the association between exposures and small-for-gestational-age (SGA), low birthweight (LBW), and Fenton growth *z*-score outcomes, adjusting for confounders. Living in a high climate vulnerability index neighborhood was tested as an effect modifier. During preconception, increases in heat stress and WF measures were associated with higher odds of SGA. Living in the most climate-vulnerable neighborhoods during preconception significantly modified and nearly doubled the odds of SGA with exposure to heat stress. Similarly, heat stress and WF exposure in trimester-specific time periods were associated with adverse birth outcomes. Conversely, third-trimester exposures were associated with lower odds of LBW. Throughout pregnancy, two measures of infant size (SGA and Fenton *z*-scores) were lower among those with greater exposure to multiple WF exposures. This study highlights how living in more climate-vulnerable neighborhoods significantly modifies the effect of heat stress on SGA, suggesting that the increasing adaptation capacity of communities may strengthen climate change resilience.

## Graphical abstract

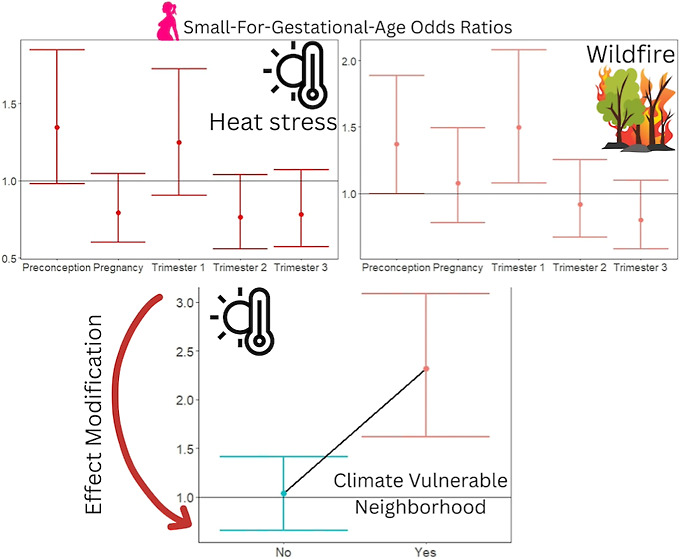

## 1 Introduction

Climate change is intensifying extreme weather events, and global warming due to human activities, namely emissions of greenhouse gases, is unprecedented, with projected increases of 1.5 °C global annual average surface temperature by 2050, according to the Intergovernmental Panel on Climate Change 2021 report.^1^ As a result, wildfires (WFs) are increasing in frequency and severity throughout the world, with extreme temperatures and heat stress projected to also increase.^2–4^ The last 10 years were the highest on record for frequency of WFs and extreme temperatures, especially in California.^5^ Climate hazards such as heat waves, droughts, and WFs are not only increasing in frequency and magnitude separately^6,7^ but also more likely to occur concurrently or consecutively as compound events, challenging the adaptive capacity of communities and human resiliency to withstand their effects.^8–10^ These dependencies lead to more complex and correlated patterns of coexposures that require careful consideration to accurately assess their cascading health risks.^8–11^

Increasingly, calls for protecting vulnerable communities and susceptible populations, including pregnant women, fetuses, and children, against climate hazards and increasing their adaptive capacity and resilience are being made.^12–15^ It is well established that poor air quality is associated with numerous adverse health outcomes, and pregnant women and fetuses are especially vulnerable to the effects of ambient air pollution, including WF smoke.^16,17^ Several studies have found associations between prenatal exposure to PM_2.5_ (particulate matter less than 2.5 μm in aerodynamic diameter) and smaller birthweight and gestational age.^18–26^ Air pollutants, such as PM_2.5_ and polycyclic aromatic hydrocarbons, released during WF events are thought to cross the placental barrier, impacting the growth of the fetus.^19,20,27–29^ However, studies examining the effects of WF smoke during pregnancy are limited and have yielded mixed results. Additionally, because of decreased ability to thermoregulate, pregnant women and fetuses are susceptible to the effects of heat stress, with exposure to extreme temperatures during pregnancy being positively associated with low birthweight (LBW) and preterm birth.^30–32^ Extreme heat is a known teratogen, and high temperatures have been shown to cause fetal damage.^32,33^ The second and third trimesters have been identified as critical windows of exposure during pregnancy for heat stress, but few studies have investigated earlier windows in preconception.^34–36^ Preconception is also a critical window of time during which gametogenesis occurs, and the effects of air pollution during preconception may lead to adverse fetal and neonatal developmental outcomes.^19,28,37^ Even less is known about critical windows of exposure for WF smoke effects on adverse growth and birth-related outcomes.

Exposure assessment challenges are thought to largely contribute to this inconsistent or limited literature. WF smoke plumes are highly dynamic in their emissions, fate, and transport patterns, and their chemical composition is complex and highly variable.^38–42^ This creates challenges in accurately assessing WF-specific source contributions to ground-level air quality and separating their health impacts from the overall air pollution mixture.^39,41,43,44^ Combustion heat amplifies plume rise and, among other things, results in widely variable vertical profiles, where smoke can be detected aloft but not reach ground level.^45^ Vertical distribution is not captured by the hazard mapping system (HMS) smoke density contours,^46^ which rely on satellite imagery (top-down views) and expert annotation and are most commonly used in WF studies or for issuing health advisories.^47,48^ Despite these limitations, HMS has been shown to correlate well with elevated ground-level PM_2.5_ concentrations.^49–51^ WF smoke plumes also get transported over long ranges, impacting not only local communities but also regions further downwind. As the plume ages, it undergoes photochemical reactions and forms secondary, oxidized pollutants such as ozone and secondary organic aerosol, which are potentially more toxic.^41,52^ Similarly for heat stress, temperature (*T*) alone does not sufficiently capture the physiological impact of thermal comfort, while measures of apparent *T* are an improvement, they tend to highly correlate with *T*, making their effects difficult to disentangle.^53^ Wet bulb globe temperature (WBGT)^54^ is thought to correlate the most with physiological responses to heat stress and the body’s ability to dissipate metabolic heat, which also increases during pregnancy,^31,55^ especially when outdoors in direct sunlight.^56^ Gridded meteorological models and data sets are increasingly offering highly spatiotemporally resolved data to assess exposure to WBGT; however, their various strengths and limitations for exposure and health studies are just starting to be better understood.^57^

Finally, the National Academies of Sciences, Engineering, and Medicine^58^ and others have noted that climate change health impacts are highly inequitable, impacting socially disadvantaged and historically marginalized populations the most—a phenomenon termed the “climate gap.” ^59–64^ In California, historically redlined and environmentally burdened neighborhoods often exist in urban heat islands (UHIs) or developed areas with limited vegetation and natural spaces. UHIs exacerbate exposure to extreme heat and challenge adaptation overall and during WFs.^60^ Furthermore, factors such as local preparedness for evacuation, resources for adaptation (e.g., vehicle access, cooling centers, etc.), and resilience-promoting factors such as neighborhood sociocultural ties and cohesion can determine climate vulnerability at very local scales. The climate vulnerability index (CVI)^65^ was recently developed to capture climate change risks and vulnerabilities nationwide at a census-tract level to inform these questions. Yet very few studies have investigated the contribution of joint prenatal exposure to WF smoke and extreme temperatures on adverse birth outcomes in a health disparities population and whether living in an UHI or climate-vulnerable neighborhood modifies these associations.

Our study aims to disentangle the health effects of WF smoke exposure and heat stress in a health disparities pregnancy cohort in Los Angeles, California, living in some of the most highly environmentally burdened and climate-vulnerable neighborhoods. We investigated impacts of these independent and joint (two) exposures on small-for-gestational-age (SGA), LBW, and Fenton growth *z*-scores outcomes, and whether effects varied by living in an UHI and by neighborhood climate vulnerability. To inform potential future work on critical exposure windows, we also investigated effects during preconception, during overall pregnancy, and in specific trimesters.

## 2 Methods

### 2.1 Study Population

Maternal And Developmental Risks from Environmental and Social stressors (MADRES) is an ongoing prospective pregnancy cohort study of primarily low-income and Hispanic mothers in Los Angeles County designed to study the impacts of environmental, psychosocial, and behavioral risk factors on maternal and infant health. Beginning in 2015, pregnant women with gestation prior to 30 weeks were recruited from four prenatal care providers in Los Angeles County. These institutions mostly serve medically underserved populations and consist of one private obstetrics and gynecology practice, two nonprofit community health clinics, and one county hospital clinic.^66^ Over 1000 participants were recruited into MADRES, but for this analysis, only the first 713 births in active participants occurring between 2016 and 2020 were included at the time of funding this specific WF smoke and heat stress climate project.

Eligibility for recruitment included the following: (1) over 18 years of age, (2) less than 30 weeks gestation, and (3) fluent in either English or Spanish. Exclusion criteria consisted of (1) inability to give informed consent due to physical, mental, or cognitive disability, (2) HIV-positive status, (3) multiple gestation, or (4) current incarceration.

### 2.2 Exposure Assessment

#### 2.2.1 Residential Histories

Daily residential histories are assembled and geocoded for all participants starting two years before birth up until the latest follow-up time point. These integrate information from residential history questionnaires, contact, and tracking information and capture temporal and spatial uncertainty in location ascertainment. These daily timelines serve as the basis for all spatiotemporal exposure assessments and clearly delineate preconception, gestational, and postpartum time windows for all mother–child pairs.

We defined preconception as the one month preceding the gestation day, pregnancy (based on exact gestational age in days and trimesters as first (0–13 weeks), second (14–28), and third (from 28+)) and used these exposure window definitions to summarize the heat stress and WF smoke exposure metrics described below.

#### 2.2.2 Meteorology and Heat Stress

##### 2.2.2.1 Surface Meteorological Data

The Abatzoglou gridded 4 × 4 km^2^ gridMET model^67^ was used to link daily estimates of outdoor minimum and maximum temperature (*T*), minimum and maximum relative humidity (RH), precipitation, wind speed and direction, and downward shortwave radiation at the residential grid.

##### 2.2.2.2 Wet Bulb Globe Temperature

Daily WBGT in °C was approximated from daily average *T* and RH (calculated from daily minimums and maximums in gridMet since hourly data were not available), shortwave radiation, and wind speed using previously described equations.^68,69^

##### 2.2.2.3 Daily Maximum Heat Index

Daily maximum heat index (DMHI) was calculated using the Rothfusz regression equation for heat index^70^ based on daily minimum RH and maximum *T*, with appropriate adjustments when RH < 13% and *T* is between 80 and 112 °F (26.7–44.4 °C) or RH > 85% or *T* is between 80 and 87 °F (26.7–30.6 °C) based on the National Oceanic and Atmospheric Association (NOAA) National Weather Service recommendations^71^ and converted to °C for use in exposure and health models.

#### 2.2.3 Wildfire Smoke and Ambient Air Pollution

##### 2.2.3.1 California Department of Forestry & Fire Protection (CalFIRE) Wildland Fire Statistics

CalFIRE^72^ provides location (latitude and longitude), burn area (acres), and start/end dates of every wildland fire within CA. This information was obtained for our study period and region (southern California, defined as 35° latitude to the north and −115° longitude to the east) and linked by time (active dates for each fire burn event, where multiple fires could be burning on the same day) to MADRES participant timelines. We calculated several exposure metrics that captured number, magnitude (size in acres), duration (days), and proximity to active WFs in southern California that overlapped with MADRES preconception and pregnancy periods (2016–2020).

Specifically, for each exposure window, we calculated the number of days with at least one active WF and the mean distance-weighted acres burned of all WFs (accounts for multiple burns on any given day and weights closer and larger fires more heavily).

##### 2.2.3.2 Hazard Mapping System Smoke Density

Daily, census block group level WF smoke densities (light, medium, and heavy) corresponding to 0–10, 10–21, and 22+ μg/m^3^ of ground-level PM_2.5_, respectively, were linked to residential locations on timelines based on models developed by Vargo^73,74^ that have been used in several studies.^48,75^ Similarly to CalFire data, we calculated the following measures: number of days with light, medium, or heavy smoke in each time period.

##### 2.2.3.3 Hybrid Single-Particle Lagrangian Integrated Trajectory Estimates of Ground-Level Wildfire Smoke-Related PM_2.5_ Concentrations

Daily estimates of WF smoke-related PM_2.5_ (WF-PM_2.5_) concentrations were modeled using the Hybrid single-particle Lagrangian integrated trajectory (HYSPLIT) model and fire emission factors by Sonoma Technology, Inc. Primary emissions were calculated using fire radiative power (FRP) from the moderate resolution imaging spectroradiometer instrument onboard Aqua and Terra satellites, similar to our earlier effort.^76^ Emission factors from the fire energetics and emissions research version 1.0 (FEER.v1) model^77^ were applied to FRP to estimate emissions. HYSPLIT was run using 12 × 12 km^2^ gridded meteorological data from the North American Mesoscale Forecasting System up through 2021 (the latest follow-up time point on MADRES timelines at the time). Emissions from all fires in California and all large fires (>1000 acres) throughout the western US and portions of Mexico and Canada were calculated using ecoregion-specific per-detect area estimates, per the 2014 National Emissions Inventory. To reduce computational requirements, all hotspots were clustered within 0.05° using density-based DBSCAN methodology,^78^ and their emissions were summed. Daily emissions were temporally distributed using the WRAP hourly time profile,^79^ which estimates minimal emissions (<0.01%) between 8 PM and 9 AM, with peak emissions at 4P M (17% of total). The 100 m surface layer concentrations were used as ground-level PM_2.5_ from WF smoke. We also defined a high WF-PM_2.5_ day where HYSPLIT WF-PM_2.5_ ≥ 0.0409 μg/m^3^ (50th percentile based on daily data) and calculated the number of high WF-PM_2.5_ days within each exposure period of interest.

##### 2.2.3.4 Ambient 8 h Maximum Ozone Concentrations

Daily maximum 8 h ambient ozone (O_3_) concentrations (ppb) were estimated at residences using inverse distance square weighted spatial interpolation of measurements from the dense regulatory monitoring network in southern California, available from the EPA Air Quality System as described earlier.^66,80^ Average concentrations for each exposure period of interest were then calculated and used to adjust for possible confounding in WF-PM_2.5_ health models, as this is the only exposure metric that directly modeled WF contributions to ground-level air quality. This was done since O_3_ can form through secondary photochemical processes as WF smoke plumes get transported and aged, and WF-PM_2.5_ concentrations modeled with HYSPLIT capture only primary particulate emissions and not secondarily formed particles or gases.

#### 2.2.4 Urban Heat Island Index

The UHI index was assigned at the residence based on a microscale model of air temperatures in urban areas compared to nearby upwind rural areas developed by the CalEPA for California.^81^ It captures the temperature differential in °C introduced by urbanization, which removes natural sinks of temperature like vegetation and replaces them with hardscaped surfaces and heat generators (like air conditioning units and vehicles), in units of degree-hours per day. We calculated a binary variable for living in an UHI based on ≥75th percentile cutoff of the mean UHI index during pregnancy (which properly weights the time spent at each location if the participant lived at more than one residence).

#### 2.2.5 Climate Vulnerability Index

CVI developed by the Environmental Defense Fund is composed of four baseline vulnerabilities, including health (e.g., access to care), social/economic (e.g., housing), infrastructure (e.g., transportation), and environment (e.g., pollutant sources), as well as three climate change risks, including health (e.g., *T*-related deaths), social/economic (e.g., economic productivity losses), and extreme events (e.g., frequency of droughts an- WFs). It is the first nationwide census-tract-level measure to comprehensively capture the joint occurrence and impact of climate hazards and of factors that increase vulnerability or lower community resilience to climate change.^82^ For testing interactions in health models, we created a binary variable to represent living in a highly climate-vulnerable neighborhood based on participants’ mean CVI ≥ 75th percentile during pregnancy.

### 2.3 Outcomes

Birthweight data were obtained from abstraction of medical records following delivery. LBW was defined as a delivery with birthweight <2500 g. Growth-for-gestational age *z*-scores are calculated based on birthweight using the Fenton growth charts^83^ and categorized into SGA, appropriate-for-gestational-age, and large-for-gestational age.

### 2.4 Covariates

Detailed questionnaires were collected at repeated time points during pregnancy, starting from the first trimester through postdelivery. Questionnaire data include but are not limited to smoking history, personal health history, pregnancy history (parity, gestational age, weight gain), demographics (age, race, ethnicity), and air conditioning use in the home during pregnancy.

### 2.5 Statistical Analysis

#### 2.5.1 Descriptive Statistics

Descriptive statistics were calculated to summarize participant characteristics, exposures, and outcome distributions. Bivariate analysis and directed acyclical graphs were used to screen for potential confounders and identify minimum adjustment sets, respectively. Correlations were calculated between covariates to inform and avoid collinearity. Pearson’s correlation coefficients were calculated for continuous variables, Cramer’s V correlation coefficients were calculated for categorical variables, and an ANOVA test was used for screening associations between categorical and continuous variables.

#### 2.5.2 Linear Regression Models

Several generalized linear models were used to investigate the independent and joint associations between WF smoke and heat stress exposures and the outcomes. Continuous outcomes included Fenton *z*-scores, and binary outcomes included SGA and LBW.

All models were adjusted for Hispanic ethnicity, smoking history (ever, never, current), parity (1st child, second child, third or more), and use of air conditioning (yes/no) in the home in pregnancy. LBW models were further adjusted for gestational age. Having an SGA baby and Fenton *z*-scores were further adjusted for diabetes during pregnancy. Diabetes status was defined as a binary variable which combined three categories of glucose intolerance, gestational diabetes, and chronic diabetes from electronic medical record data. Glucose intolerant was defined as a borderline (120–<140 mg/Dl) or positive (140–<200 mg/Dl) glucose challenge test (GCT) and a follow-up oral glucose tolerance test (OGTT) with at least one positive score, or a positive GCT result but no follow-up OGTT. Gestational diabetes was defined as a positive GCT result and a follow-up OGTT with 2 or more positive scores, or a positive GCT result and a physician diagnosis of GDM in the medical record, or a high-positive GCT result. Models examining WF-PM_2.5_ as the primary exposure of interest were further adjusted for 8 h maximum O_3_, as described earlier.

We first ran single exposure models for each WF smoke and heat stress variable, and we then ran joint (two) exposure models by including the WF smoke and heat stress variables that were most consistently and strongly associated with the outcomes in the single exposure models. All effect estimates (for continuous outcomes) and odds ratios (for binary outcomes) were scaled to a one standard deviation (SD) increase in exposure for each time period (i.e., SD specific to time period). A p-value <0.05 was considered as a threshold for statistical significance.

#### 2.5.3 Interactions

Effect modification was tested for WBGT and WF days using interaction terms for infant sex, living in an UHI, and living in a highly climate-vulnerable neighborhood with SGA and Fenton *z*-scores. We considered a limited set of heat stress and WF exposures and outcomes for testing interactions based on the previous associations observed in single exposure models. We considered a *p*-value <0.05 as a significant interaction.

## 3 Results

### 3.1 Descriptive Statistics

Descriptive statistics of participant characteristics and outcomes are listed in Table 1. Our MADRES study population consisted of primarily Hispanic (74%) women and lower-income women (42% of participants reported a household income of less than $30,000/year). Most mothers reported never smoking (87.5%), did not have chronic or gestational diabetes nor were they glucose intolerant (64%), and for the majority, this was not their first child (52.4%). The majority of births in this cohort were full term (89.6%) and normal birthweight (93%).

**Table 1:** Select Characteristics of Study Participants and Outcome Distributions (*n* = 713)

characteristic	*n* (%)
Child Sex	
male	359 (50.3)
female	351 (49.2)
missing	3 (0.4)
Diabetes Status	
no diabetes	465 (64.0)
glucose intolerant	137 (19.2)
gestational diabetes	61 (8.6)
chronic diabetes	34 (4.8)
missing	16 (2.2)
Maternal Hispanic Ethnicity	
yes	525 (73.6)
no	146 (20.5)
missing	42 (5.9)
Smoking History	
never	624 (87.5)
ever	54 (7.6)
current	19 (2.7)
missing	16 (2.2)
Parity	
1st child	217 (30.4)
2nd child	187 (26.2)
3rd or more child	187 (26.2)
missing	122 (17.1)
Air Conditioner Use in the Home in Pregnancy	
yes	363 (50.9)
no	254 (35.6)
missing	96 (13.5)

birth outcomes	*n* (%)
SGA	
yes	48 (6.8)
no	650 (92.1)
missing	8 (1.1)
LBW	
yes	41 (5.8)
no	657 (93.1)
missing	8 (0.01)
	mean (SD)
Fenton *z*-score (unitless)	–0.08 (0.87)

Figure 1 illustrates the large day-to-day variability in WF incidences (occurrence, number, size) during the study period. On any given day in the preconception and pregnancy period (total 216,141 person-days from 713 participants), participants experienced a mean number of 1.16 (SD 1.65, min 0, max 11) active WF burnings and a mean of 176.3 (SD 690.7, min 0, max 8106.0) distance-weighted acres of WFs burning. Around 48% of all person-days were active WF days in southern California (102,980 person-days). Daily WF-PM_2.5_ concentrations ranged from 0 to 1160.5 μg/m^3^ (mean 1.02, SD 8.09 μg/m^3^).

**Figure 1:**
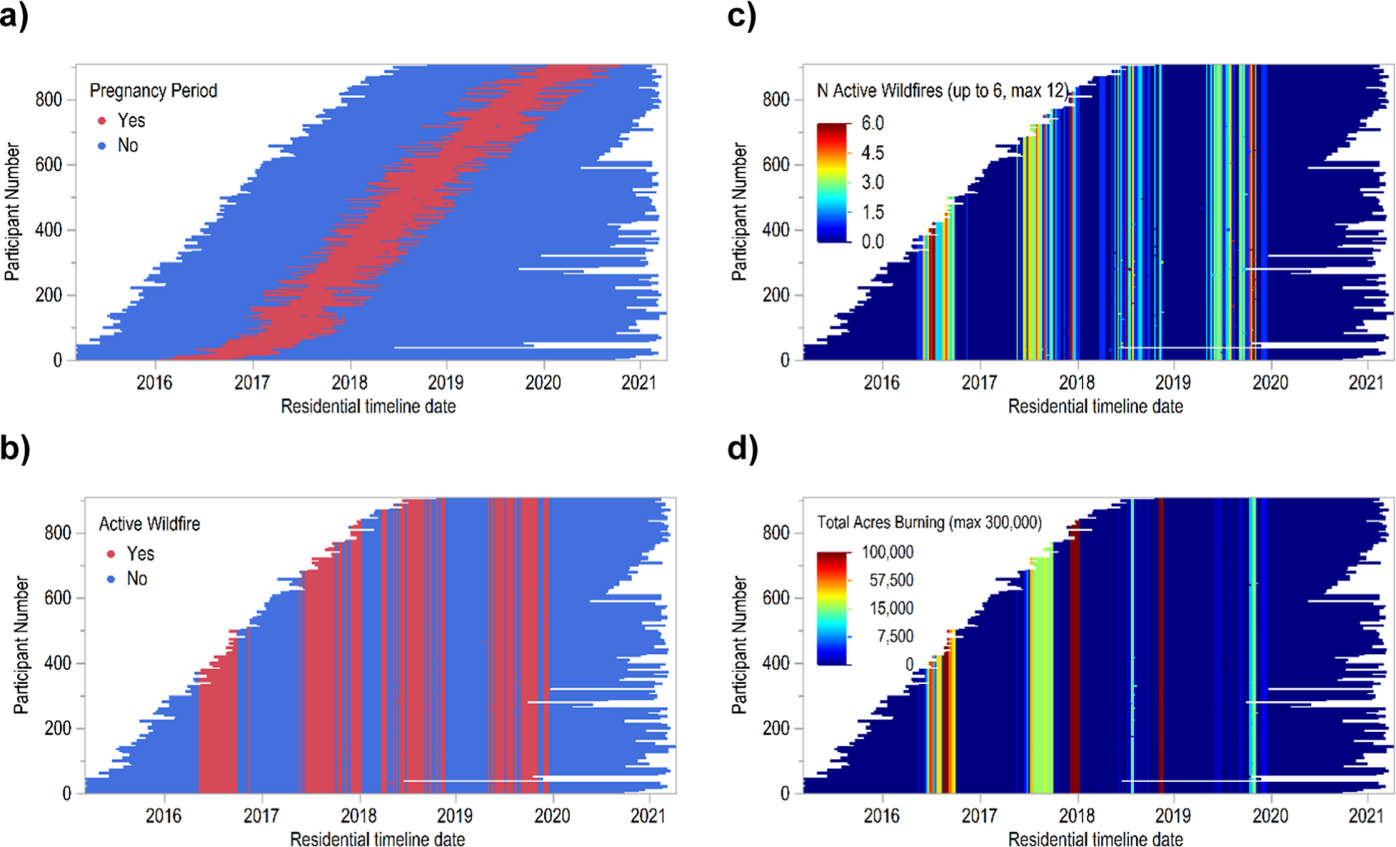
Daily residential timelines of MADRES participants illustrating overlap between (a) days of pregnancy periods, (b) days with at least one active WF in southern California, (c) number of simultaneously burning active WFs (colored up to 6, maximum is 12) on a given day, and (d) total acres burning (colored up to 100,000 acres, maximum is ∼300,000 acres) on a given day.

Table 2 presents summary statistics for exposures averaged during pregnancy and preconception and trimesters. On average in pregnancy, women were exposed to 131 active WF days and 135 days with high WF-PM_2.5_ concentrations. Mean WBGT and DMHI were 17 and 23 °C, respectively. The average distance-weighted area burned of WFs was 179 acres, and women were exposed to an average of 13.4 light, 0.95 medium, and 0.8 heavy smoke density days. Large variations in the averages across trimesters were not observed for these exposures. However, lower values were observed during preconception, likely due to the shorter time frame captured during that period.

**Table 2:** Distribution of WF and Heat Stress Exposures in Different Preconception and Prenatal Windows

	mean (SD) by time period				
exposure	preconception	pregnancy	trimester 1	trimester 2	trimester 3
Heat Stress					
WBGT (°C)	16.8 (3.8)	17.1 (1.3)	17.0 (3.5)	17.2 (3.4)	17.1 (3.5)
DMHI (°C)	23.2 (4.1)	23.5 (1.6)	23.3 (3.6)	23.6 (3.5)	23.5 (3.7)
WF Smoke by Data Source					
CalFIRE					
N active WF days	13.7 (11.7)	130.7 (42.5)	43.2 (28.9)	48.9 (30.1)	38.9 (28.0)
distance-weighted area burned (acres)	160.2 (558.1)	178.7 (207.3)	189.4 (397.9)	173.8 (362.0)	173.0 (393.1)
HYSPLIT					
WF-PM_2.5_ concentration (μg/m^3^)	0.8 (1.4)	1.0 (0.9)	0.9 (1.1)	1.0 (1.2)	1.2 (2.6)
high WF-PM_2.5_ days	14.6 (7.8)	135.4 (26.1)	43.7 (16.9)	49.6 (16.8)	42.6 (16.3)
HMS					
light density smoke days	1.5 (2.9)	13.4 (7.8)	4.3 (5.7)	4.9 (6.0)	4.2 (5.5)
medium density smoke days	0.10 (0.4)	0.95 (1.4)	0.27 (0.8)	0.35 (0.9)	0.34 (1.0)
heavy density smoke days	0.1 (0.3)	0.8 (1.2)	0.2 (0.4)	0.3 (0.5)	0.3 (1.0)

Pregnancy-wide temperature exposures (DMHI and WBGT) were the most highly correlated with each other (Pearson *r* = 0.83), while DMHI correlations with WF measures were higher than those of WBGT. Medium- and heavy-density smoke days were highly correlated (*r* = 0.68), while light-density smoke days were most correlated with high WF-PM_2.5_ concentration days (*r* = 0.67), suggesting light-density smoke days best capture ground-level primary WF smoke impacts on air quality. The number of active WF days in pregnancy was most highly correlated with DMHI (*r* = 0.68) and WBGT (*r* = 0.65), followed by the number of high WF-PM_2.5_ days (*r* = 0.58) and the number of light-density smoke days (*r* = 0.56). Remaining Pearson correlations between all exposures in pregnancy are shown in Supporting Information Figure S2.

### 3.2 Single Exposure Model Results

Figure 2 presents the odds ratios (for binary outcomes) or effect estimates (for continuous outcomes) and their respective 95% CIs for all single exposure models, which are also summarized below. Supporting Information Figure S1 shows these same results on the same *Y*-axis scale to compare across outcomes. For WF-PM_2.5_ models, adjustment for 8 h maximum O_3_ did not markedly change the effect estimate of WF-PM_2.5_.

**Figure 2:**
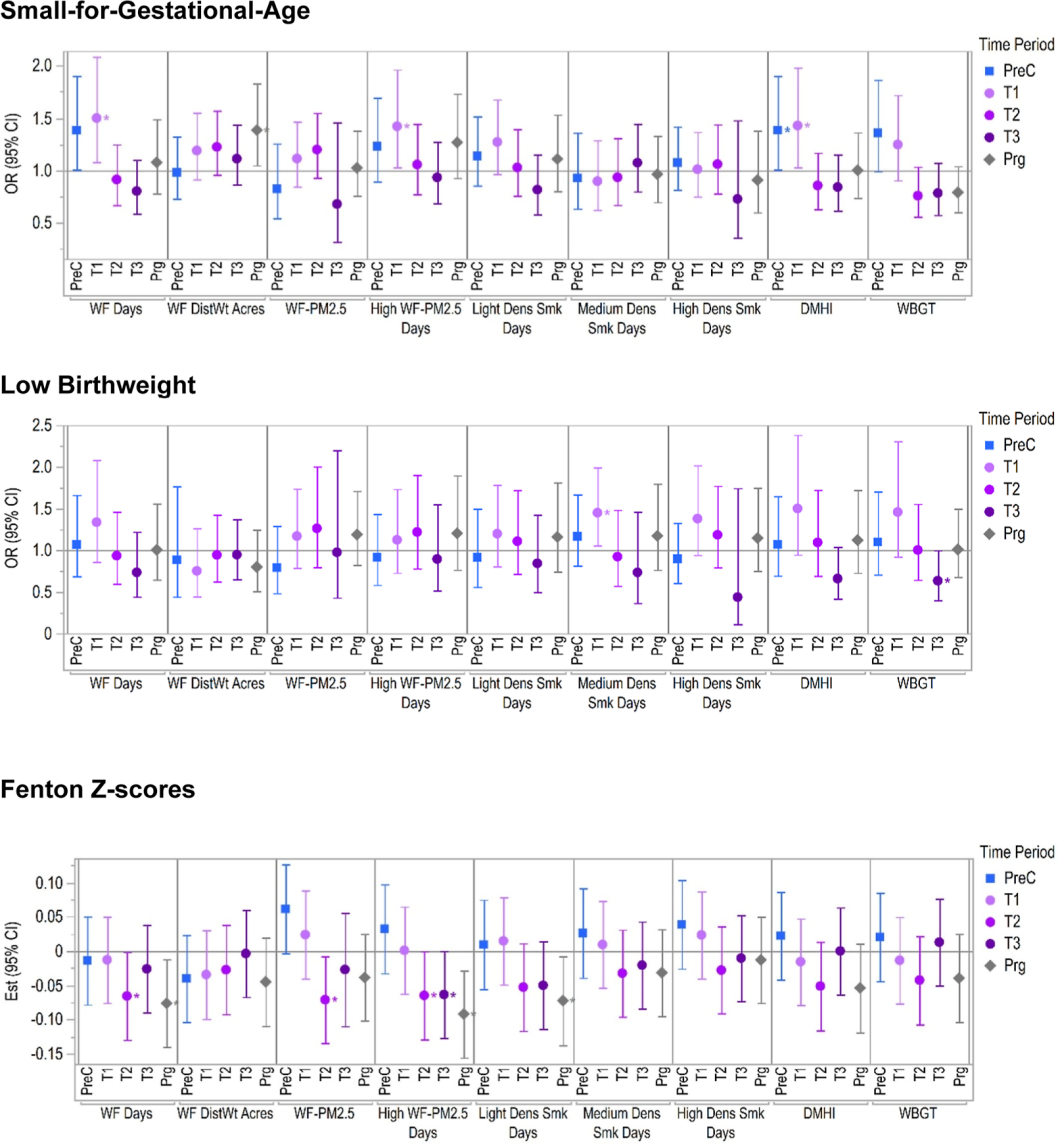
Results of single exposure models showing odds ratios (for binary outcomes) and effect estimates (for continuous outcomes) with their respective 95% confidence intervals per exposure and time period, scaled to an SD change in the exposure. *P*-values <0.05 are indicated with an asterisk.

Because missingness patterns in adjustment covariates and in exposures differed across time periods, the final number of observations included in each model across outcomes and for the same outcome varied from a minimum of 575 to a maximum of 704 (Supporting Information Table S3).

#### 3.2.1 Small-for-Gestational-Age

During preconception, an increase in odds of having an SGA baby was observed for a SD increase in DMHI (OR: 1.37 (95% CI: 1.00, 1.89)).

Similarly, in the first trimester, odds of SGA increased with exposure to DMHI (1.43 (1.03, 1.99)), number of high WF-PM_2.5_ days (1.42 (1.03, 1.96)), and number of active WF days (1.50 (1.08, 2.08)). Across pregnancy, an SD increase in the distance-weighted area of WFs burned was also associated with greater odds of SGA (1.38 (1.04, 1.82)).

#### 3.2.2 Low Birthweight

The odds of LBW were significantly increased with greater exposure to medium density smoke days in the first trimester (OR: 1.45 (95% CI: 1.06, 1.99)). However, an SD increase in WBGT exposure in the third trimester decreased odds of LBW (0.63 (0.40, 0.99)).

#### 3.2.3 Fenton Growth-for-Gestational-Age *z*-Scores

Throughout pregnancy and during the second trimester, there were significant decreases in Fenton *z*-score values associated with an SD increase in several WF-related exposures. In pregnancy, significant decreases in Fenton *z*-scores were observed with the number of high WF-PM_2.5_ days (est: −0.09 (95% CI: −0.16, −0.03)), active WF days (−0.08 (−0.14, −0.01)), and light density smoke days (−0.07 (−0.14, −0.01)). Effects were similar, but slightly smaller, for the number of high WF-PM_2.5_ days in the second and third trimesters and active WF days in the second trimester.

### 3.3 Joint Exposure Model Results

To further explore joint (two) exposure models, we chose the exposures and outcomes that showed the most consistent associations across all time periods in single exposure models, which were the number of active WF days and WBGT with SGA and Fenton *z*-scores. Results of joint exposure models mutually adjusting for both WF days and WBGT are shown for SGA and Fenton *z*-scores in Figure 3.

**Figure 3:**
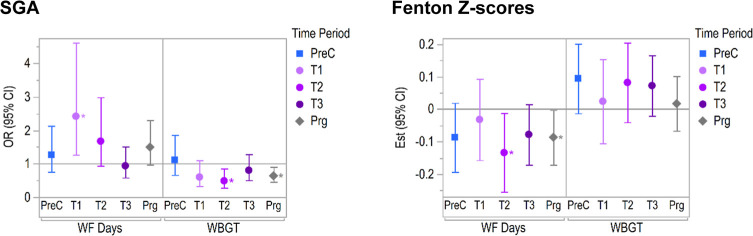
Results of joint (two) exposure models showing mutually adjusted odds ratios (for SGA) and effect estimates (for Fenton *z*-scores) and their respective 95% confidence intervals for exposure to the number of active WF days and WBGT across different time periods, scaled to an SD change in the exposure. *P*-values <0.05 are indicated with an asterisk.

Briefly, the association between SGA and number of active WF days in preconception was attenuated and no longer significant compared to the single exposure models; however, in the first trimester, it increased to OR: 2.4 (95% CI: 1.25, 4.50) compared to 1.50 (1.08–2.08). Whereas the effect of WBGT on odds of SGA in the second trimester and the pregnancy became more significantly negative (versus negative but null in single-exposure models).

Decreased Fenton *z*-scores during the second trimester and pregnancy-wide exposure of WF days remained significant and became more negative (pregnancy-wide est: −0.09 (95%CI: −0.17, −0.003); second trimester: −0.14 (−0.26, −0.02)).

### 3.4 Effect Modification Results

Based on our earlier findings, and similarly to how we chose to look at the number of active WF days and WBGT in joint (two) exposure models with SGA and Fenton *z*-scores, we further investigated potential interactions between these exposures and the baby’s sex, living in a UHI, and living in a more climate-vulnerable neighborhood. To limit the number of tests, we investigated exposures only in the preconception and pregnancy-wide periods for interactions.

There were no significant interactions with the baby’s sex, living in a UHI, or number of WF days with either outcome. However, we found that during preconception, A positive and significant association was observed between SGA and WBGT for mothers living in neighborhoods in the top 75th percentile of the CVI score, reflecting a more climate-vulnerable neighborhood. Figure 4 illustrates the marginal predicted probabilities of SGA from these fully adjusted models, showing clearly diverging predicted probabilities of SGA at greater exposure levels, increasing in the high CVI group. These results were also confirmed in a fully stratified model as a sensitivity analysis (not shown, living in the top 75th percentile CVI: WBGT OR: 2.32 (1.62, 3.03) versus living in the lowest 75th percentile of CVI scores: WBGT 1.04 (0.66, 1.41)).

**Figure 4:**
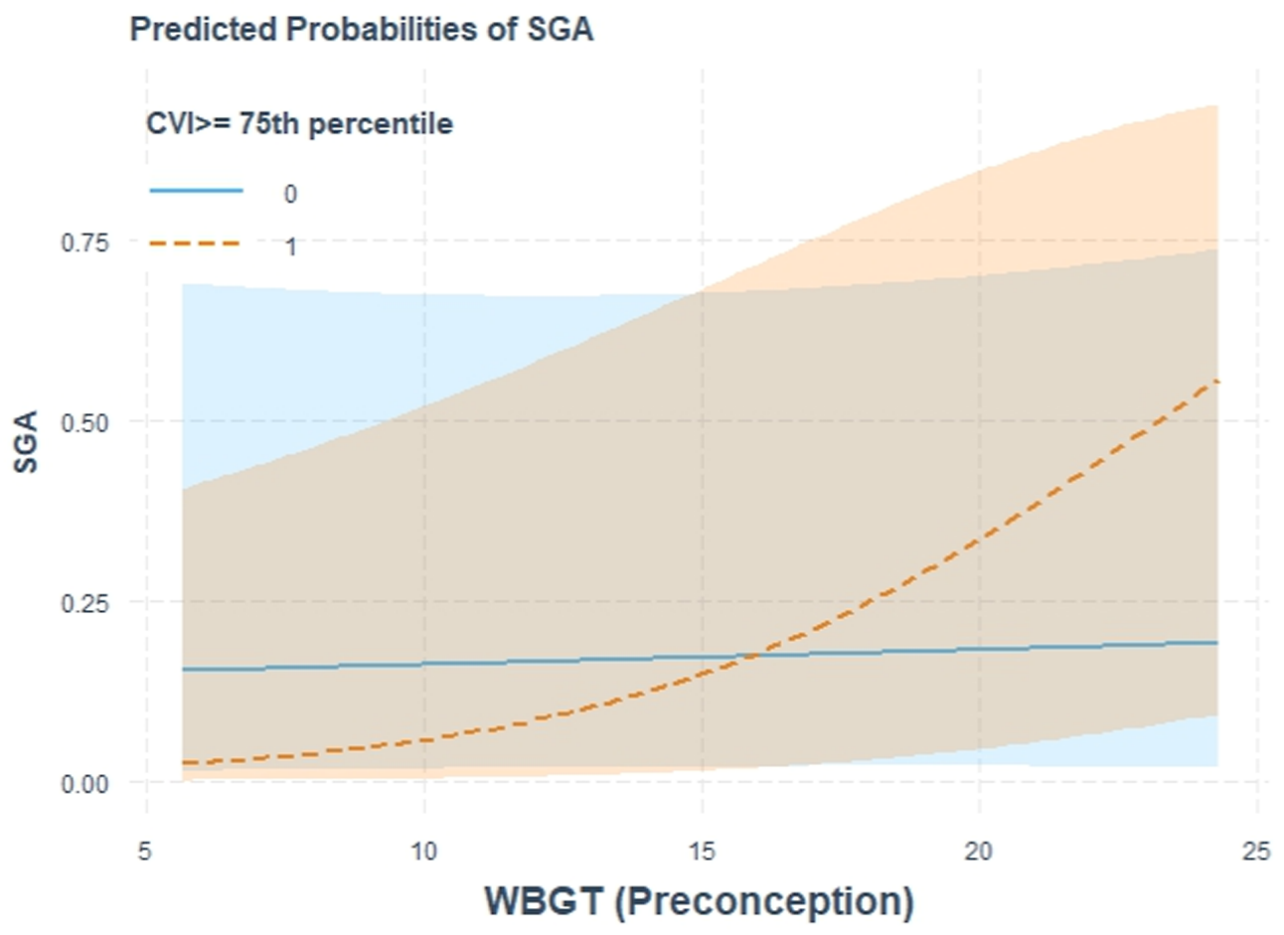
Marginal predicted probabilities of SGA from fully adjusted* single exposure models testing for interaction of exposure to WBGT with living in more climate-vulnerable neighborhoods during pregnancy (defined based on 75th percentile of CVI scores). *Adjusted for diabetes, Hispanic race, smoking history, parity, and AC use during pregnancy.

No significant interactions were observed with the Fenton *z*-scores.

## 4 Discussion

In this analysis, we examined the effects of several measures of heat stress and WF smoke ranging in complexity and meant to comprehensively capture different aspects of exposure on SGA, LBW, and Fenton growth-for-gestational-age *z*-scores in the MADRES pregnancy cohort. Participants are primarily low-income Hispanic women living in Los Angeles, CA, in some of the most environmentally burdened and climate-vulnerable neighborhoods. We also examined joint (two) exposures, the importance of different time windows of exposure starting from preconception, and the modifying effect of living in more climate-vulnerable neighborhoods that experience more climate hazards and have greater social vulnerabilities and lower resources to deal with or recover from them, generally thought to lower climate adaptive capacity and resilience. Overall, we found strong and consistent associations for increased exposures to DMHI and the number of WF days experienced during preconception and the first trimester with odds of SGA. Fenton *z*-scores also significantly decreased with a greater number of days of WF smoke experienced during the entire pregnancy and in the second trimester, and these associations were consistent across several WF metrics, including active WF days, high WF-PM_2.5_ days, light density smoke days, and concentrations of WF-PM_2.5_. For those living in more climate-vulnerable neighborhoods, the effects of WBGT on the odds of SGA were also significantly higher than those in less climate-vulnerable neighborhoods, as captured with the CVI.

We found greater odds of SGA with exposure to WF days and to DMHI, and the preconception period and first trimester were particularly important windows of exposure. It has been well established that prenatal exposure to air pollutants is linked to adverse birth outcomes; however, limited studies report on the contribution of WF smoke exposure, and even fewer have looked at the preconception period. Studies have found that exposure to air pollution during preconception had positive associations with SGA,^84,85^ while others found no associations.^86,87^ A previous study found that a large proportion of pregnant people in California are exposed to PM_2.5_ from WF smoke during preconception, as well as during the first and second trimesters.^88^ A systematic review of eight studies with almost two million births found the evidence for WF smoke exposure in pregnancy and risk of SGA to be inconclusive, although it was only examined in only two of the eight studies. While there was some evidence for later pregnancy exposures to be associated with lower birth weight and preterm birth, the authors noted the need for more comprehensive studies.^89^

WF days and DMHI exposures were highly correlated in the 1 month preconception period, and their effects were similar, making it difficult to tell which exposure may be driving the SGA associations. However, throughout pregnancy, the distance-weighted WF area burned was most strongly associated with SGA, suggesting WF smoke exposure may be driving these effects in preconception. Similarly, in joint (two) exposure models, when adjusted for WBGT, the number of WF days in the first trimester remained significantly associated with odds of SGA. However, given the moderately elevated correlation between WF days and WBGT across pregnancy (*r* = 0.65), these results, while supportive of stronger effects of WF smoke exposure compared to heat stress, should be interpreted with caution. Compound climate events, defined as co- or consecutively occurring climate hazards that are increasingly frequent due to the highly connected and interdependent nature of the physical processes driving them, are creating more complex patterns of correlated climate-related exposures.^8,11^ These necessitate new and advanced methods to accurately capture their cascading and possibly synergistic impacts on health, as independent or compound events, and joint “co-exposure” models are just a simple first step in that direction. During California’s 2020 fire season, 42% of the population were estimated to have experienced at least a one-time exposure to both extreme temperatures and high PM_2.5_ from WF smoke.^90^ Another study found the effects of high PM_2.5_ and high temperature exposure were greater than their individual effects when experienced together.^91^

Fenton *z*-scores were also consistently lower with pregnancy-wide and second-trimester WF smoke exposures (WF days, high WF-PM_2.5_ days, light-density smoke days, and WF-PM_2.5_ concentrations). Effect estimates for these exposure measures were very similar but most significantly negative for WF days and high WF-PM_2.5_ days suggesting local southern California fires most directly impacting ground-level concentrations of WF smoke were more sensitive than satellite-observed smoke density contours from NOAA HMS which are known to have poor vertical resolution (i.e., are not capable of distinguishing smoke at ground level vs aloft).^51^ Several other studies have also found WF smoke exposure throughout pregnancy and in mid to late pregnancy to be associated with adverse birth outcomes such as LBW and preterm birth.^47,48,92–96^ However, our findings are contradictory to a recent San Francisco study that found second-trimester exposure to WF-specific PM_2.5_ and to days with WF-specific PM_2.5_ > 5 μg/m^3^ to be associated with greater birthweight for gestational age.^93^ This could be due to the notably different WF smoke exposure assessment strategies used in both studies, with zip-level statistically derived estimates versus residential level physical dispersion modeled estimates of WF-PM_2.5_ concentrations. Gan et al.^97^ also noted risk estimates of WF smoke exposure differed based on the method for estimating WF-PM_2.5_ for the same outcome in an analysis of cardiopulmonary hospital admissions. They compared chemical transport models to kriged surface PM_2.5_ measurements and hybrid models that combine both with satellite aerosol optical depth data. They attribute discrepancies to the method in which nonWF-sourced PM_2.5_ is estimated and parsed out of the overall PM_2.5_ and air quality mixture in the various models. This is a critically important point, as proper source attribution (of particles and gases, both primary and secondary) is still very much complicated by the complex nonlinear chemistry involved in WF smoke emissions and plume transport, and simple differencing approaches are most likely introducing large biases and uncertainty in current epidemiological analyses of WF smoke effects on health.

As for exposure to heat stress, while many studies have found extreme temperatures (low or high) and thermal stress in pregnancy to be associated with adverse birth outcomes,^18,36,98,99^ we only found preconception and first-trimester effects of DMHI on greater odds of SGA, which seemed to be driven by WF smoke exposure, and third-trimester WBGT on lower odds of LBW, which is counterintuitive. When adjusted for WF smoke exposure in joint (two) models (as WF days), WBGT exposure also became negatively associated with odds of SGA in midpregnancy (second trimester) and pregnancy-wide. The contrary to expected results is not unique to our study and may suggest that exposure aversion behavior is at play. Pregnant individuals might modify their behaviors to stay indoors or in air-conditioned spaces more and avoid higher outdoor temperatures or heavier or more perceivable WF smoke, voluntarily or due to public health directives and warnings issued for vulnerable populations to protect themselves when heat waves or WFs occur, especially later in the pregnancy, as also found and discussed in Magzamen et al.^100^ Early work in our cohort using personal PM_2.5_ monitoring in the third trimester of pregnancy is starting to show some evidence of this exposure aversion behavior, where the agreement between ambient vs personal PM_2.5_ concentrations declines as smoke density increases and potentially gets more perceivable to the layperson. Whereas, in no or light WF smoke conditions, measured personal PM_2.5_ exposures correlate better with ambient PM_2.5_ concentrations.^101^ This could also explain why light-density smoke days were more negatively associated with adverse birth outcomes in our study than medium or heavy smoke days.

Understanding how behaviors change and which actions are protective against WF smoke exposure, especially for vulnerable populations like children, pregnant or health-compromised individuals, or the elderly, is critically important given how frequent and long WF seasons are expected to become. How well WF smoke infiltrates indoors and how the different chemical components in the particle and gas phases behave in indoor environments, which have very complex chemistry,^102^ are crucial to understanding how effective exposure mitigation strategies like indoor air “cleaning” or filtration can be. Xiang et al.^103^ found that staying indoors during WF episodes is not protective enough against excessive exposure to WF smoke, with PM_2.5_ infiltration factors ranging from 0.3 to 0.8 (reflecting how well outdoor PM_2.5_ infiltrates the building envelope and gets indoors), and portable air cleaner efficacy ranged from 48 to 78% in seven residences in the Seattle, Washington area during a 2020 WF episode. More importantly, Li et al.^104^ showed in an experimental test house injected with smoke that window opening and portable air cleaners were less efficient at permanently removing volatile organic compounds (VOCs), or chemicals introduced by WF smoke into indoor air, since these can partition onto particles and surfaces and get re-emitted into air when equilibrium conditions shift to favor gas partitioning. However, cleaning by vacuuming, mopping, and dusting of floors, walls, and ceilings was more effective at reducing these WF-related VOC gas-phase concentrations over the longer term, since it effectively removed these surface reservoirs. This work highlights the fact that the WF smoke mixture of health concern is much more chemically diverse than just particles, not just in the outdoor environment but also as it enters indoor environments and contacts people (i.e., becomes an exposure).

Finally, understanding vulnerability to WF smoke, heat stress, and other climate-related hazards and exposures is key to targeting interventions and investments to where they are most needed to reduce health disparities.^58,105^ We found that the association between WBGT and odds of SGA was close to double in preconception among those living in the most climate-vulnerable neighborhoods of our study, highlighting the importance of understanding cumulative impacts of climate-related environmental exposures in the context of human, social, and infrastructural factors that shape climate adaptive capacity and resilience. Living in a UHI and the number of WF days were not significant interactions. Our findings are in line with the National Academies of Sciences, Medicine, and Engineering recommendations for the United States Environmental Protection Agency’s Office of Research and Development to consider cumulative impacts and systems thinking in its science framework, which recognizes the interconnected nature of environmental processes, societal factors, and multiple pathways in which stressors influence human health rather than thinking of single exposures in isolation.^106^

Our study has several strengths and limitations. First, we were able to investigate multiple time points starting from preconception using highly resolved daily residential timelines and WF smoke and heat stress measures. The different WF smoke exposure estimates that we used each have their own strengths and weaknesses in terms of their ability to capture direct impacts of WF smoke on air quality and exposure. Southern California CalFire data (WF days, distance, and size) track most closely with public safety alerts or notifications in our study region around local or more proximal fires while they are burning. As such, it could be well suited at capturing elements of exposure most directly influencing behaviors. It also provided some of the most consistent associations in health analyses, despite not directly modeling smoke. The HYSPLIT model provided more accurate source apportioned estimates of WF-PM_2.5_ from primary WF smoke emissions; however, it does not capture the chemical composition of smoke, especially the differential impacts of very fresh vs aged smoke, which could contain more toxic compounds.^107^ And finally, while the NOAA HMS smoke density contours potentially capture both fresh and long-range transport of smoke, they do not resolve vertical distribution of smoke or capture ground-level impacts and overall were not the most sensitive measures in health analyses. Second, our cohort is a highly characterized, lower-income population of pregnant women living in some of the most environmentally burdened and climate-vulnerable neighborhoods of California. As such, our findings are highly relevant for understanding health disparities and cumulative impacts related to climate change and adaptation.

A limitation in our study was the small sample sizes for some of our outcomes, which may have been underpowered to detect some significant associations. Most of the babies in this study population were born at full term, were not SGA, and were normal birthweight. Another limitation was that we did not account for where mothers worked and time spent outside of their home. This leads to potential measurement error in those women who spend a substantial amount of time outside their homes. Further, we acknowledge that multiple comparisons were made with results including different pregnancy time points and different outcomes, with 8–10 of our significant findings potentially being due to chance; however, our number of significant findings exceeded our type 1 error rate of 0.05 (with 15 significant associations and effect estimates in our single exposure models), lessening the likelihood that these are due to chance.

In conclusion, we found that WF smoke and heat stress exposures during preconception and early pregnancy showed positive, significant associations with SGA, and WF smoke exposure throughout pregnancy was associated with reduced Fenton *z*-scores. This is one of the first studies to show that living in more climate-vulnerable neighborhoods is significantly associated with heat, suggesting that increasing the adaptation capacity of communities may strengthen climate change resilience. Future work using personal monitoring data will help us better understand patterns of exposure measurement error during WFs,^101^ and using distributed lag models will strengthen our ability to identify critical windows of exposure, while also considering the combination of factors driving vulnerability and resilience to climate change.

## Supplementary Material



## Notes

Results of single and joint exposure models with odds ratios and effect estimates with their respective 95% confidence intervals per exposure and time period (scaled to a SD change in the exposure), number of observations used in models and SDs of each exposure used to scale results, Pearson correlation matrix and heat map of pregnancy-wide heat stress, WF smoke exposures and CVI, and distribution of WF and heat stress exposures for the select outcome of SGA (PDF)
