# Supplementary material for: Adverse Birth Outcomes Associated with Heat Stress and Wildfire Smoke Exposure During Preconception and Pregnancy
Source: Environ Sci Technol. 2025 Jun 18;59(25):12458–71. doi: 10.1021/acs.est.4c10194 (PMC12224298; doi:10.1021/acs.est.4c10194)
Supplement: Supplementary file 1 [file es4c10194_si_001.pdf]

SUPPORTING INFORMATION

Adverse Birth Outcomes Associated with Heat Stress and Wildfire Smoke Exposure during Preconception and Pregnancy

Roxana Khalili*,[1] Yisi Liu,[1] Yan Xu,[1] Karl O'Sharkey,[1] Nathan Pavlovic,[2] Crystal McClure,[2] Fred Lurmann,[2] Tingyu Yang,[1] Xinci Chen,[1] Mario Vigil,[1] Brendan Grubbs,[3] Layla Al Marayati,[3] Deborah Lerner,[4] Nathana Lurvey,[4] Carmen J. Marsit,[5] Jill Johnston,[1] Theresa M. Bastain,[1] Carrie V. Breton,[1] Shohreh F. Farzan,[1] Rima Habre[1,6]

Affiliations

[1] Department of Population and Public Health Sciences, Keck School of Medicine, University of Southern California, Los Angeles, CA 90032, United States

[2] Sonoma Technology, Inc., Petaluma, CA 94954, United States

[3] Department of Obstetrics and Gynecology, Keck School of Medicine, University of Southern California, Los Angeles, CA 90033, United States

[4] Eisner Health, Los Angeles, CA 90015, United States

[5] Gangarosa Department of Environmental Health, Rollins School of Public Health, Emory University, Atlanta, GA 30322, United States

[6] Spatial Sciences Institute, Los Angeles, CA 90089, United States

Contents

Table S1. Results of single exposure models showing odds ratios (for binary outcomes) and effect estimates (for continuous outcomes) with their respective 95% confidence intervals per exposure and time period, scaled to an SD change in the exposure. S1

Table S2. Results of joint (two) exposure models showing mutually adjusted odds ratios (for SGA) and effect estimates (for Fenton Z-scores) and their respective 95% confidence intervals for exposure to number of active wildfire days and wet bulb globe temperature across different time periods, scaled to an SD change in the exposure. S2

Table S3. Number of observations used in models and standard deviations of each exposure used to scale results. S3

Figure S1. Pearson correlation matrix and heat map of pregnancy wide heat stress, wildfire smoke exposures, and climate vulnerability index. S4

Table S4. Distribution of wildfire and heat stress exposures for the select outcome of Small-for-Gestational-Age. S5

Table S1. Results of single exposure models showing odds ratios (for binary outcomes) and effect estimates (for continuous outcomes) with their respective 95% confidence intervals per exposure and time period, scaled to an SD change in the exposure.

		Pre-conception				Trimester 1				Trimester 2				Trimester 3				Pregnancy			
Outcome	Exposure	OR or Est	Lower 95% CI	Upper 95% CI	P-value	OR or Est	Lower 95% CI	Upper 95% CI	P-value	OR or Est	Lower 95% CI	Upper 95% CI	P-value	OR or Est	Lower 95% CI	Upper 95% CI	P-value	OR or Est	Lower 95% CI	Upper 95% CI	P-value
Fenton Z-score	WF Days	-0.01	-0.08	0.05	0.7009	-0.01	-0.08	0.05	0.6970	-0.07	-0.13	0.00	0.0404	-0.03	-0.09	0.04	0.4402	-0.08	-0.14	-0.01	0.0186
	WF DistWt Acres	-0.04	-0.10	0.02	0.2160	-0.03	-0.09	0.03	0.3649	-0.03	-0.09	0.04	0.3917	0.00	-0.07	0.06	0.8964	-0.04	-0.11	0.02	0.1842
	WF-PM2.5	0.06	0.00	0.13	0.0631	0.02	-0.04	0.09	0.4623	-0.07	-0.13	-0.01	0.0286	-0.03	-0.11	0.06	0.5273	-0.04	-0.10	0.02	0.2327
	High WF-PM2.5 Days	0.03	-0.03	0.10	0.2959	0.00	-0.06	0.07	0.9693	-0.07	-0.13	0.00	0.0451	-0.07	-0.13	0.00	0.0440	-0.09	-0.16	-0.03	0.0039
	Light Dens Smk Days	0.01	-0.05	0.08	0.7489	0.01	-0.05	0.08	0.6607	-0.05	-0.12	0.01	0.0990	-0.05	-0.11	0.01	0.1225	-0.07	-0.14	-0.01	0.0255
	Medium Dens Smk Days	0.03	-0.04	0.09	0.4290	0.01	-0.05	0.07	0.7792	-0.03	-0.10	0.03	0.3118	-0.02	-0.08	0.04	0.5425	-0.03	-0.10	0.03	0.3299
	High Dens Smk Days	0.04	-0.02	0.11	0.1982	0.02	-0.04	0.09	0.4752	-0.03	-0.09	0.04	0.3823	-0.01	-0.07	0.05	0.7462	-0.01	-0.08	0.05	0.6783
	DMHI	0.02	-0.04	0.09	0.4700	-0.02	-0.08	0.05	0.6131	-0.05	-0.12	0.01	0.1120	0.00	-0.06	0.06	0.9805	-0.06	-0.12	0.01	0.0949
	WBGT	0.02	-0.04	0.09	0.513	-0.01	-0.08	0.05	0.6548	-0.04	-0.11	0.02	0.1861	0.01	-0.05	0.08	0.6681	-0.04	-0.10	0.02	0.2118
Small-for-Gestational-Age	WF Days	1.37	1.00	1.89	0.0508	1.50	1.08	2.08	0.0164	0.92	0.67	1.26	0.5991	0.80	0.59	1.10	0.1701	1.08	0.78	1.49	0.6443
	WF DistWt Acres	0.98	0.73	1.33	0.9113	1.17	0.90	1.53	0.2340	1.23	0.96	1.57	0.1083	1.11	0.86	1.44	0.4093	1.38	1.04	1.82	0.0242
	WF-PM2.5	0.81	0.53	1.24	0.3353	1.11	0.84	1.46	0.4584	1.20	0.93	1.55	0.1562	0.69	0.32	1.47	0.3305	1.03	0.76	1.38	0.8587
	High WF-PM2.5 Days	1.22	0.89	1.67	0.2258	1.42	1.03	1.96	0.0333	1.06	0.77	1.45	0.7170	1.03	0.75	1.42	0.864	1.27	0.93	1.74	0.1287
	Light Dens Smk Days	1.13	0.85	1.51	0.406	1.27	0.96	1.68	0.0899	1.03	0.76	1.40	0.8288	0.82	0.58	1.16	0.2592	1.12	0.81	1.54	0.4999
	Medium Dens Smk Days	0.92	0.63	1.36	0.6864	0.90	0.62	1.29	0.5634	0.93	0.67	1.31	0.6936	1.07	0.80	1.44	0.6401	0.97	0.70	1.34	0.832
	High Dens Smk Days	1.06	0.80	1.40	0.6845	1.01	0.75	1.37	0.9357	1.06	0.78	1.44	0.7102	0.74	0.36	1.49	0.3943	0.91	0.60	1.38	0.6677
	DMHI	1.37	1.00	1.89	0.0499	1.43	1.03	1.99	0.0325	0.86	0.63	1.18	0.3492	0.84	0.61	1.15	0.2784	1.01	0.74	1.37	0.9626
	WBGT	1.35	0.98	1.85	0.0653	1.25	0.91	1.72	0.1750	0.76	0.56	1.04	0.0870	0.78	0.57	1.07	0.1239	0.79	0.60	1.05	0.1022
Low Birth Weight	WF Days	1.07	0.69	1.66	0.7707	1.34	0.86	2.09	0.2006	0.93	0.6	1.46	0.7659	0.73	0.44	1.22	0.2337	1	0.65	1.56	0.9867
	WF DistWt Acres	0.88	0.44	1.77	0.7279	0.75	0.45	1.26	0.2792	0.94	0.62	1.43	0.7827	0.95	0.65	1.37	0.7708	0.8	0.51	1.25	0.3211
	WF-PM2.5	0.79	0.49	1.29	0.3476	1.17	0.79	1.74	0.4374	1.26	0.79	2	0.3236	0.97	0.43	2.2	0.9512	1.19	0.82	1.71	0.357
	High WF-PM2.5 Days	0.92	0.58	1.44	0.7019	1.12	0.73	1.74	0.5964	1.22	0.78	1.9	0.3833	0.89	0.51	1.55	0.69	1.2	0.76	1.9	0.4277
	Light Dens Smk Days	0.91	0.56	1.5	0.7202	1.2	0.8	1.79	0.3739	1.11	0.71	1.72	0.6488	0.84	0.5	1.43	0.5221	1.16	0.74	1.81	0.5202
	Medium Dens Smk Days	1.17	0.82	1.67	0.4005	1.45	1.06	1.99	0.0218	0.92	0.57	1.48	0.7385	0.73	0.37	1.46	0.3783	1.17	0.76	1.8	0.47
	High Dens Smk Days	0.9	0.61	1.33	0.5914	1.38	0.94	2.02	0.0979	1.18	0.79	1.77	0.4094	0.44	0.11	1.75	0.2415	1.15	0.75	1.75	0.5276
	DMHI	1.07	0.69	1.65	0.7606	1.5	0.95	2.38	0.0849	1.09	0.69	1.72	0.7026	0.66	0.42	1.04	0.0715	1.12	0.73	1.72	0.6061
	WBGT	1.1	0.71	1.71	0.6748	1.46	0.92	2.31	0.1075	1	0.65	1.56	0.9902	0.63	0.4	1	0.0492	1.01	0.68	1.49	0.9662

Table S2. Results of joint (two) exposure models showing mutually adjusted odds ratios (for SGA) and effect estimates (for Fenton Z-scores) and their respective 95% confidence intervals for exposure to number of active wildfire days and wet bulb globe temperature across different time periods, scaled to an SD change in the exposure.

		Pre-conception				Trimester 1				Trimester 2				Trimester 3				Pregnancy			
**Outcome**	**Exposure**	**OR or Est**	**Lower 95% CI**	**Upper 95% CI**	**P-value**	**OR or Est**	**Lower 95% CI**	**Upper 95% CI**	**P-value**	**OR or Est**	**Lower 95% CI**	**Upper 95% CI**	**P-value**	**OR or Est**	**Lower 95% CI**	**Upper 95% CI**	**P-value**	**OR or Est**	**Lower 95% CI**	**Upper 95% CI**	**P-value**
Fenton Z-s	WF Days	-0.09	-0.19	0.02	0.1095	-0.03	-0.15	0.10	0.6563	-0.14	-0.26	-0.02	0.0275	-0.08	-0.17	0.02	0.1019	-0.09	-0.17	0	0.0429
	WBGT	0.09	-0.01	0.2	0.0855	0.02	-0.11	0.15	0.7743	0.08	-0.04	0.2	0.1868	0.07	-0.02	0.17	0.1280	0.02	-0.07	0.1	0.7253
SGA	WF Days	1.27	0.76	2.13	0.3990	2.37	1.25	4.50	0.0084	1.69	0.95	3.01	0.0740	0.94	0.59	1.51	0.8073	1.5	0.97	2.3	0.0663
	WBGT	1.11	0.66	1.85	0.6990	0.59	0.32	1.09	0.0930	0.49	0.28	0.85	0.0120	0.8	0.49	1.29	0.3627	0.64	0.46	0.91	0.0122

Table S3. Number of observations used in models and standard deviations of each exposure used to scale results.

		Pre-Conception	Trimester 1	Trimester 2	Trimester 3	Pregnancy	Pre-Conception	Trimester 1	Trimester 2	Trimester 3	Pregnancy
**Outcome**	**Exposure**	**N obs**					**SD of Exposure**				
Fenton Z-score	WF Days	679	683	686	687	688	11.7	28.9	30.1	28	42.5
	WF DistWt Acres	679	683	686	687	688	558.1	397.9	362	393.3	207.3
	WF-PM2.5	676	680	684	682	686	1.4	1.1	1.2	2.6	0.9
	High WF-PM2.5 Days	679	683	686	687	688	7.8	16.9	16.8	16.3	26.1
	Light Dens Smk Days	679	683	686	687	688	2.9	5.7	6	5.5	7.8
	Medium Dens Smk Days	679	683	686	687	688	0.4	0.8	0.9	1	1.4
	High Dens Smk Days	679	683	686	687	688	0.3	0.4	0.5	1	1.2
	DMHI	683	685	687	688	688	4.1	3.6	3.5	3.7	1.6
	WBGT	683	685	687	688	688	3.8	3.5	3.4	3.5	1.3
Small-for-Gestational-Age	WF Days	679	683	686	687	688	11.7	28.9	30.1	28	42.5
	WF DistWt Acres	679	683	686	687	688	558.1	397.9	362	393.3	207.3
	WF-PM2.5	677	681	685	683	687	1.4	1.1	1.2	2.6	0.9
	High WF-PM2.5 Days	679	683	686	684	688	7.8	16.9	16.8	16.3	26.1
	Light Dens Smk Days	679	683	686	687	688	2.9	5.7	6	5.5	7.8
	Medium Dens Smk Days	679	683	686	687	688	0.4	0.8	0.9	1	1.4
	High Dens Smk Days	679	683	686	687	688	0.3	0.4	0.5	1	1.2
	DMHI	683	685	687	688	688	4.1	3.6	3.5	3.7	1.6
	WBGT	683	685	687	688	688	3.8	3.5	3.4	3.5	1.3
Low Birth Weight	WF Days	695	699	702	703	704	11.7	28.9	30.1	28	42.5
	WF DistWt Acres	695	699	702	703	704	558.1	397.9	362	393.3	207.3
	WF-PM2.5	693	697	701	699	703	1.4	1.1	1.2	2.6	0.9
	High WF-PM2.5 Days	695	699	702	703	704	7.8	16.9	16.8	16.3	26.1
	Light Dens Smk Days	695	699	702	703	704	2.9	5.7	6	5.5	7.8
	Medium Dens Smk Days	695	699	702	703	704	0.4	0.8	0.9	1	1.4
	High Dens Smk Days	695	699	702	703	704	0.3	0.4	0.5	1	1.2
	DMHI	699	701	703	704	704	4.1	3.6	3.5	3.7	1.6
	WBGT	699	701	703	704	704	3.8	3.5	3.4	3.5	1.3

Figure S1. Pearson correlation matrix and heat map of pregnancy wide heat stress, wildfire smoke exposures, and climate vulnerability index.

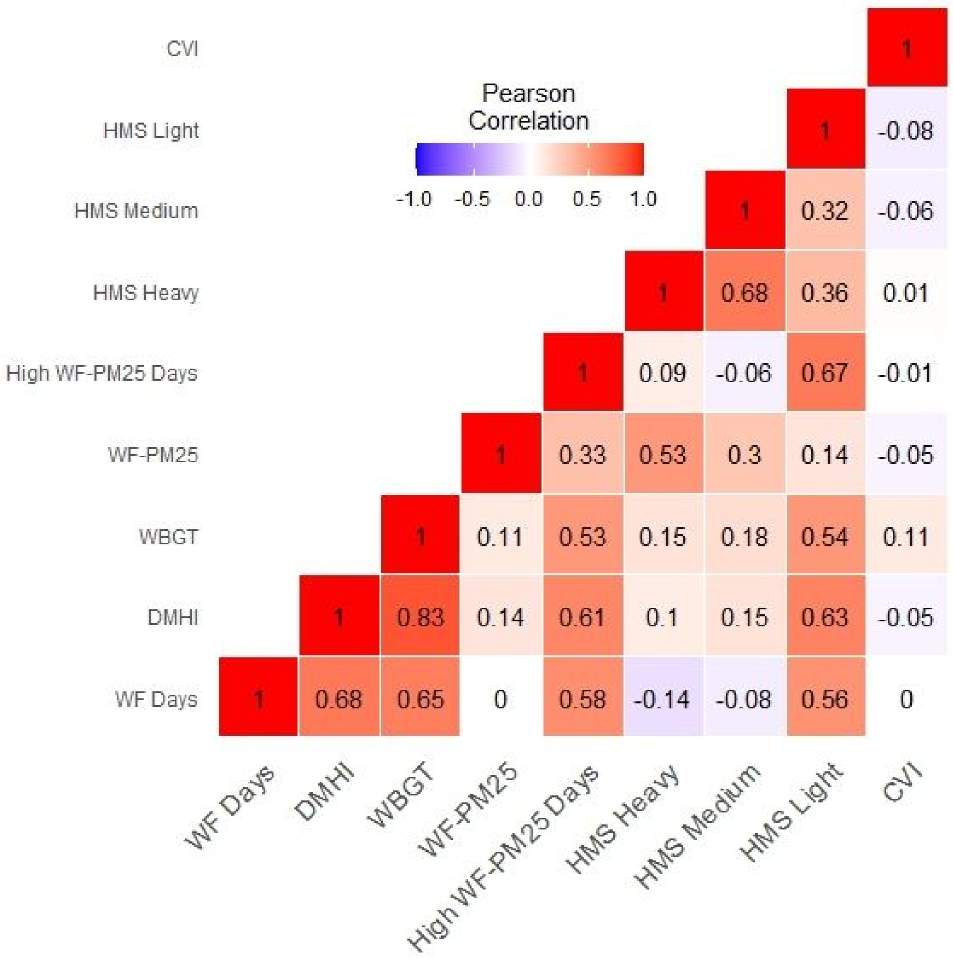

Table S4. Distribution of wildfire and heat stress exposures for the select outcome of Small-for-Gestational-Age.

Outcome	Exposure	Yes (mean (SD))	No (mean(SD))
Small-for-Gestational-Age	WF Days	134.7 (42.7)	130.6(42.5)
	WF DistWt Acres	253.8 (233.0)	173.4 (204.6)
	WF-PM2.5	1.07 (0.47)	1.05 (0.90)
	High WF-PM2.5 Days	142.1 (23.9)	135.1 (26.3)
	Light Dens Smk Days	14.5 (7.7)	13.4(7.8)
	Medium Dens Smk Days	0.87 (1.4)	0.96 (1.4)
	High Dens Smk Days	0.72 (0.71)	0.82 (1.2)
	DMHI	23.5 (1.2)	23.5 (1.6)
	WBGT	16.8 (1.4)	17.1 (1.3)
